# James Greig Smith

**Published:** 1897-06-05

**Authors:** 


### JAMES GREIG SMITH, M.A., M.B.

It is with great regret that we have to record the death of James Greig Smith, the well-known Bristol surgeon, which took place last Friday at his residence in Clifton. There is no doubt that he was one of the most brilliant surgeons of the day, a keen observer, full of originality in his operative proceedings, and possessing in a high degree that deftness of finger and delicacy of manipulation which are so essential in modern surgery. It is only a little more than twenty years since he came to Bristol to fill the office of assistant house surgeon, and he has died in his forty-fourth year. Nevertheless, he has built up for himself not only a great consulting practice, but what it is no exaggeration to describe as a world-wide fame as an abdominal surgeon. He was at the time of his death surgeon to the Bristol Royal Infirmary, and Professor of Surgery in the University College, Bristol. In 1883 he, along with Mr. L. M. Griffiths, started the *Bristol Medico-Chirurgical Journal*, which he and his colleague at first edited, a duty which on his resigning the post was undertaken by a small committee. He was the author of many important contributions to medical and surgical literature. His most generally known work is his book on "Abdominal Surgery," of which a fifth edition is now in the press, a book which is widely accepted as an authoritative exposition of a subject which its author did much to illuminate, and has already been translated into French and German for foreign publishers. But Greig Smith was not only a great surgeon. He was one of those men who naturally take a leading position in whatever they take up, either in the way of work or play. He was thorough and practical in every respect, and the diversity of his knowledge, skill, and accomplishments was a surprise to his numerous friends, to whom he was a delightful companion, and one from whom accurate information on various topics could be acquired. He seemed to have a tolerably full knowledge of many subjects, a proof that his reading had been wide and deep, and that his memory was exceedingly good. He could converse well; argue well; speak well at meetings or at public dinners. He was an excellent sculptor and fine draughtsman, as indicated by his many and beautiful anatomical specimens, and by other examples of his artistic taste. He was a clever architect, and could prepare a plan of a building, and carry out the work if need be. He was a good sportsman, and at his pleasant little retreat at Burnet he was occasionally joined by a select shooting party from Bristol. He had a passion for golfing, and was the life and leading spirit of the club to which he belonged. He was fond of yachting. Deceased married, about 12 years ago, Miss Ashby, member of a West Indian family. Mr. Greig Smith was one of those brilliant and skilful men seldom to be met with; and his death causes a vacancy in his profession in the West of England which it will be difficult to fill. Devotion to his duties is believed to have brought on the malady from which he died. Strong though he was, two attacks of rheumatic fever made inroads on his constitution, and pneumonia, from which he suffered about 12 months ago, further weakened him. On Monday he went to see a patient in Wiltshire, and had to drive a long distance. He then felt pains of pleurisy, which he thought were symptoms of rheumatism, and paid little heed to them. In the evening he dined at the house of a friend, and visited the Nursing Home. On Tuesday inflammation of the lungs set in, and Drs. Shaw and James Swain were called to attend him. No anxiety was felt about his condition, however, until Thursday, when there was an increase of dangerous symptoms, which rapidly developed, and proved fatal on Friday morning. He was buried on Tuesday last at Redland Green, amid the large gathering of medical colleagues, nurses, and friends, who, despite the inclement weather, had assembled, many coming long journeys by rail to join with his sorrowful relatives in the long farewell.

Greig Smith was a man whom to know was to honour, respect, and love. Punctilious in honour himself, he was impatient of anything which appeared to savour of insincerity. To children he displayed a gentle and winning sweetness, which was most attractive in a man of muscular physique and powerful presence, and amongst the large number of those who most deeply feel his all too-early death are his many little friends. We understand that his friends and colleagues hope to perpetuate his memory by some memorial connected with his work at the Bristol Royal Infirmary.

